# Influence of Oil Viscosity on Hysteresis Effect in Electrowetting Displays Based on Simulation

**DOI:** 10.3390/mi16040479

**Published:** 2025-04-18

**Authors:** Wei Li, Linwei Liu, Taiyuan Zhang, Lixia Tian, Li Wang, Cheng Xu, Jianwen Lu, Zichuan Yi, Guofu Zhou

**Affiliations:** 1 Shenzhen Guohua Optoelectronics Technology Co., Ltd., Shenzhen 518110, China; wei.li@guohua-oet.com (W.L.) lixia.tian@guohua-oet.com (L.T.) jianwen.lu@guohua-oet.com (J.L.); guofu.zhou@m.scnu.edu.cn (G.Z.); 2 Guangdong Provincial Key Laboratory of Optical Information Materials and Technology, Institute of Electronic Paper Displays, South China Academy of Advanced Optoelectronics, South China Normal University, Guangzhou 510006, China; taiyuan.zhang@guohua-oet.com; 3 School of Information Engineering, Zhongshan Polytechnic, Zhongshan 528400, China; linwei.liu@guohua-oet.com; 4 Jihua Laboratory, 28 Huandao South Road, Foshan 528200, China; xucheng@jihualab.com; 5 Zhongshan Institute, University of Electronic Science and Technology of China, Zhongshan 528402, China; yizichuan@163.com

**Keywords:** electrowetting display (EWD), hysteresis effect, oil viscosity, simulation, contact angle hysteresis (CAH)

## Abstract

As the most promising new reflective display technology, electrowetting displays (EWDs) have the advantages of a simple structure, fast response, high contrast, and rich colors. However, due to the hysteresis effect, the grayscales of EWDs cannot be accurately controlled, which seriously restricts the industrialization process of this technology. In this paper, the oil movement process in an EWD pixel cell was simulated, and the influence of oil viscosity on the hysteresis effect was studied based on the proposed simulation model. Firstly, the cause of the hysteresis effect was analyzed through the hysteresis curve of an EWD. Then, based on the COMSOL Multiphysics simulation environment, the oil movement process in an EWD pixel cell was simulated by coupling the phase field of laminar two-phase flow and electrostatic field. Finally, based on the simulation model, the influence of oil viscosity on the hysteresis effect in an EWD pixel cell was studied. We observed that the maximum hysteresis difference in the hysteresis effect increased with the increase in oil viscosity and decreased with the decrease in oil viscosity. The oil viscosity had little effect on the maximum aperture ratio of EWD. The pixel-on response time and pixel-off response time increased with the increase in oil viscosity.

## 1. Introduction

The optical switching in electrowetting display (EWD) technology is achieved through electrical control of colored oil dynamics in pixel cells [1], where contact angle modification is induced by voltage application at solid-liquid interfaces. This principle has been applied to various fields, including microfluidic manipulation in lab-on-chip systems [2], focal length adjustment in optical devices [3], and energy conversion through liquid motion control [4]. Among these implementations, EWD-based displays are currently considered to possess the most promising commercial viability [5]. EWD technology has the characteristics of simple structure, fast response, high reflectivity, high contrast, and rich colors [6]. However, due to the rough surface of the pixel structure and the viscous resistance of the oil–water interface, the hysteresis effect will appear during the pixel driving process [7,8]. As a result, the grayscales of EWDs cannot be accurately controlled. Numerous scholars have devoted substantial efforts to mitigate the hysteresis effect.

The hysteresis effect of EWDs refers to the difference in optical response during the driving process of pixel switching [9]. It was first discovered in 2006 by Van Dijk R of Liquavista when he improved an EWD to display video content with 4 bits of grayscales and a resolution of 170 ppi [10]. In 2012, a hysteresis-free pixel switching structure was designed by adding hydrophilic patches or staircases [11]. The hysteresis effect could be better eliminated by this method, but it would reduce the aperture ratio of pixels and bring unpredictable difficulties to the manufacturing process. In the same year, the electrowetting process of electrolyte drops on smooth and rough surfaces was studied by using the lattice Boltzmann model [12]. It was observed that the hysteresis and saturation of the contact angle were more obvious on the rough surface. In 2015, a theoretical model was proposed to describe the evolution of electrowetting on substrates with contact angle hysteresis, and the relationship between the apparent contact angle, voltage, and other parameters was quantified [13]. The results show that the theory and equation based on energy balance could successfully describe the electrowetting response with obvious contact angle hysteresis. In 2017, a method and principle for improving contact angle hysteresis was given based on the improved Young’s equation [14]. The relationship between lens focal length and applied voltage was studied by using three kinds of oil with different viscosity ratios as insulating liquid. The results show that the focal length hysteresis could be reduced by reducing the oil viscosity. In 2020, a pixel structure was proposed for achieving accurate control of oil rupture position and move direction by adding additional pinning structures or spacing arrays [15,16]. This scheme could effectively reduce the hysteresis effect of EWD.

To mitigate hysteresis effects through oil viscosity modulation, using the COMSOL Multiphysics simulation platform, the oil movement process in a pixel cell was successfully simulated by coupling the laminar two-phase flow phase field and electrostatic field, and the influence of oil viscosity on the hysteresis effect in EWDs was studied based on the proposed simulation model.

## 2. Principles of Hysteresis Effect in EWDs

### 2.1. Driving Principle of EWDs

**Figure 1:**
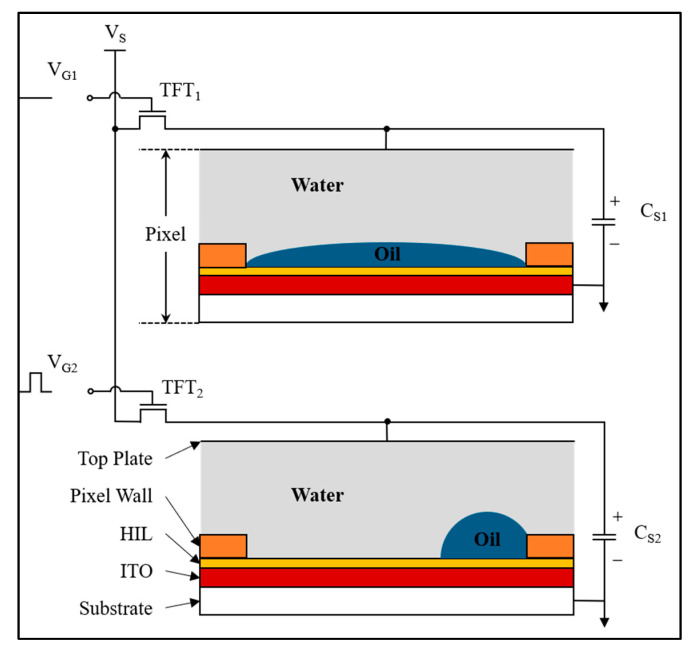
Schematic of EWD structure and driving principle. Pixels exhibit oil coloration under zero voltage, while voltage application triggers substrate exposure through interfacial reconfiguration.

An EWD panel is mainly composed of a glass substrate, indium tin oxides (ITOs), an electrode, a hydrophobic insulation layer (HIL), a pixel wall, colored oil, polar liquid, and a top plate [5,15], as shown in Figure 1. The gate control mechanism of the thin-film transistor (TFT) is realized by dual voltage regulation:

- Color display mode: When gate signal V_G1_ is biased at a low level, TFT_1_ enters the cutoff state, resulting in zero potential storage (C_S1_ = 0 V). This passive configuration enables spontaneous oil spreading to achieve full-pixel coverage through interfacial tension dominance.
- Substrate exposure mode: Activation of V_G2_ at a high-level triggers TFT_2_ conduction, enabling capacitive charging (C_S2_ = V_S_). The resultant electrostatic actuation induces oil film retraction to pixel corners, with contraction magnitude being voltage-dependently regulated for precise grayscale modulation.

Optical states correlate with oil contraction levels quantified by the aperture ratio ($W_{A}$), defined as the exposed substrate area proportion relative to total pixel area ($S_{pixel}$) [17], with computational expression provided in Equation (1).

(1) $$W_{A}(V)=\left(1-\frac{S_{oil}(V)}{S_{pixel}}\right)\times 100\%$$

where $S_{oil}$ represents the oil-covered surface area in a pixel, V represents the driving voltage applied to EWDs, and the area of pixel wall can be excluded from area calculations. Pixel walls are transparent grid structures that partition EWDs into discrete pixels.

### 2.2. Hysteresis Effect of EWDs

**Figure 2:**
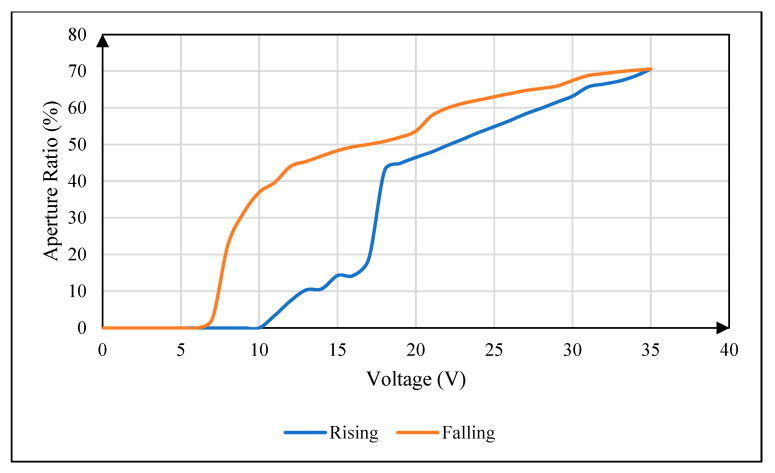
Hysteresis curve of EWD driven by conventional square wave. The blue curve is the driving voltage rising stage, and the red curve is the driving voltage falling stage.

As shown in Figure 2, it is the relationship between driving voltage and aperture ratio when the conventional square wave is used to drive an EWD panel. The blue curve is the driving voltage rising stage, and the red curve is the driving voltage falling stage. It can be seen that there is a significant difference in the aperture ratio between the driving voltage rising stage and the driving voltage falling stage, that is, hysteresis effect. Generally, the absolute value of the aperture ratio difference under the same voltage value in the rising stage and the falling stage is called the hysteresis difference, and the aperture ratio curves formed in the rising stage and the falling stage are collectively called the hysteresis curves [18].

Combined with the morphological characteristics of the hysteresis effect, through theoretical analysis and literature review [19,20,21,22], it can be seen that the main causes of the hysteresis effect in EWDs are the existence of oil rupture voltage, oil contact angle hysteresis, and oil contact angle saturation. The oil rupture voltage is caused by the fact that the electric field force is not enough to overcome the static friction and capillary force when the driving voltage is too low. Oil contact angle hysteresis is due to the inconsistency between the oil forward contact angle and the oil backward contact angle caused by dynamic friction and viscous resistance. Oil contact angle saturation is caused by the weakening of the electric field force due to charge trapping.

## 3. Numerical Methodology

The numerical method was realized by establishing and solving numerical equations. The purpose was to track the morphological changes and motion characteristics of the oil–water interface in the limited pixel cell under the action of the external electric field force. In this paper, COMSOL Multiphysics 6.0 was employed to simulate electrohydrodynamically modulated laminar biphasic flow through multiphysics coupling of hydrodynamic, interfacial, and electrostatic phenomena. Governing equations, encompassing the Cahn–Hilliard phase-field formulation, Laplace’s electrostatic equation, and Navier–Stokes momentum transport, were solved numerically using the finite element framework within a rigorously validated numerical scheme. First, the Maxwell stress tensor equation was solved by adding an electrostatic field module, and the electric field force output from the electrostatic field module was fed back to the hydrodynamic module. Then, the Navier–Stokes equation and phase field equation were solved by the hydrodynamics module according to the specified fluid boundary conditions and the solution results of the electrostatic field.

### 3.1. Governing Equations

There are two kinds of fluids in EWD pixel cells: water and oil. During calculations, the fluids are assumed to be an incompressible and incompatible Newtonian fluid, and there are no chemical reactions in the flow process. The Navier–Stokes equation with momentum conservation and the continuum equation with mass conservation are used to describe the laminar flow field [23]. The governing equations are as follows:

(2) $$\rho \left(\frac{\partial u}{\partial t}+u\cdot \nabla u\right)=-\nabla p+\nabla \cdot \left(\mu \left(\nabla u+{\left(\nabla u\right)}^{T}\right)-\frac{2}{3}u\left(\nabla \cdot u\right)I\right)+F$$

(3) $$F=F_{st}+\rho g+F_{vf}$$

where u is the fluid velocity, p is the fluid pressure, ρ is the fluid density, μ is the hydrodynamic viscosity. And each item corresponds to inertial force, pressure, viscous force and external force acting on the fluid. The external force is mainly composed of surface tension, gravity and volume force. $F_{st}$ represents surface tension (N/m^3^), g represents gravitational acceleration (m/s^2^), $F_{vf}$ is the volume force (N/m^3^).

The fluid flow in the EWD system is laminar flow ($Re=5\times 10^{-2}\ll 2000$), and there is no diffusion effect between the two fluids. Moreover, the gravity can be ignored ($Bo=1.4\times 10^{-6}\ll 1$). Therefore, electrostatic field force is the main factor causing fluid flow [24]. The parameters requiring coupling are the volume force $F_{vf}$ and the surface tension $F_{st}$ caused by the applied electric field. The electrostatic volume force (N/m^3^) can be expressed by the divergence of the Maxwell stress tensor [24].

(4) $$F_{vf}=\nabla T_{ik}$$

The Maxwell stress tensor can be expressed as Equation (5).

(5) $$T=ED^{T}-\frac{1}{2}\left(D\cdot EI\right)$$

where I is the identity matrix, E is the electric field, and D is the potential shift field.

(6) E=−∇V

(7) $$D=\varepsilon_{0}\varepsilon_{r}E$$

In the two-dimensional structural model, the Maxwell stress tensor can be expressed as Equation (8).

(8) $$T=\left[\begin{array}{cc} T_{xx} & T_{xy}\\ T_{yx} & T_{yy} \end{array}\right]$$

The stress tensor in each direction can be obtained by substituting the parameters.

(9) $$T=\left[\begin{array}{cc} \varepsilon_{0}\varepsilon_{r}E_{x}^{2}-\frac{1}{2}\varepsilon_{0}\varepsilon_{r}\left(E_{x}^{2}+E_{y}^{2}\right) & \varepsilon_{0}\varepsilon_{r}E_{x}E_{y}\\ \varepsilon_{0}\varepsilon_{r}E_{x}E_{y} & \varepsilon_{0}\varepsilon_{r}E_{y}^{2}-\frac{1}{2}\varepsilon_{0}\varepsilon_{r}\left(E_{x}^{2}+E_{y}^{2}\right) \end{array}\right]$$

The change in volume force caused by electrostatic field only occurs at the interface of two-phase fluid, and its derivative can be obtained.

(10) $$F=\left[\begin{array}{cc} \frac{\partial \left(T_{xx}\right)}{\partial x} & \frac{\partial \left(T_{xy}\right)}{\partial y}\\ \frac{\partial \left(T_{yx}\right)}{\partial x} & \frac{\partial \left(T_{yy}\right)}{\partial y} \end{array}\right]$$

After an electric field is applied, the contact angle of the three-phase contact line will be changed by the Maxwell stress. The contact angle refers to the external angle during the movement of water on the hydrophobic insulation layer, which is expressed by the Lippmann–Young equation [9]. As shown in Equation (11), where θ is the contact angle when the driving voltage is V, and $\theta_{0}$ is the contact angle when the driving voltage is 0, $\varepsilon_{0}$ and $\varepsilon_{r}$, respectively, represent the vacuum dielectric constant and the relative dielectric constant of the dielectric layer. And d is the thickness of the dielectric layer, and $\gamma_{LG}$ is the liquid–gas contact line.

(11) $$\cos\theta =\cos\theta_{0}+\frac{\varepsilon_{0}\varepsilon_{r}}{2d\gamma_{LG}}V^{2}$$

For two-phase fluid movement, the volume function method (VOF), level set method, or phase field method are usually used to track the oil–water interface [25,26]. Based on the diffusion interface model, the phase field method characterizes the interface evolution of the system through the changes in order parameters and concentration field. In the phase field theory framework of COMSOL, the comprehensive action of physical interface motion and chemical potential change is expressed by a differential equation so that the instantaneous change in interface is obtained. The phase field method does not directly track the change in the interface, but through the phase field variable ∅, it describes the fluid distance phase interface ∅=0. In the region of two fluids, the phase field variable is taken as ±1, while in the transition region of the interface, the phase field variable ∅ changes smoothly between −1 and 1. The oil–water interface is defined as a diffusion interface, the change in the oil–water interface is described by Cahn–Hilliard convection equation, and the phase field variables are obtained by solving the phase field equation [27,28,29]. The phase field governing equations are as follows:

(12) $$\frac{\partial \emptyset }{\partial t}+u\cdot \nabla \emptyset =\nabla \cdot \frac{\gamma \lambda }{\varepsilon^{2}}\nabla \psi$$

(13) $$\psi =-\nabla \cdot \varepsilon^{2}\nabla \emptyset +\left(\emptyset^{2}-1\right)\emptyset +(\frac{\varepsilon^{2}}{\lambda })\frac{{\partial f}_{ext}}{\partial \emptyset }$$

(14) $$\sigma =\frac{2\sqrt{2}\lambda }{3\varepsilon }$$

where λ is the energy density (N), and ε is the capillary width varying with the interface thickness (m). These two parameters and the surface tension coefficient σ (N/m) are described by Equation (14). γ is the mobility parameter, and the relation function between γ and ε is $\gamma =\chi \varepsilon^{2}$, χ is the mobility adjustment parameter. The phase field variable ∅ is expressed as 1 in oil and −1 in water. In order to correctly couple the electrostatic field and laminar flow field, it is necessary to track the dielectric constant, density and viscosity between diffusion interfaces. The calculation formulas are as follows:

(15) $$\rho =\rho_{1}+\left(\rho_{2}-\rho_{1}\right)\emptyset$$

(16) $$\mu =\mu_{1}+\left(\mu_{2}-\mu_{1}\right)\emptyset$$

(17) $$\varepsilon_{r}=\varepsilon_{r1}+\left(\varepsilon_{r2}-\varepsilon_{r1}\right)\emptyset$$

where ρ, μ, and $\varepsilon_{r}$ represent the density, viscosity, and dielectric constant of the fluid, respectively. The average curvature between two liquid interfaces is calculated as follows [29]:

(18) $$\kappa =2(1+\emptyset )(1-\emptyset )\frac{G}{\sigma }$$

(19) $$G=\lambda \left(-\nabla^{2}\emptyset +\frac{\emptyset \left(\emptyset^{2}-1\right)}{\varepsilon^{2}}\right)+\frac{\partial f}{\partial \emptyset }$$

where κ and G represent mean curvature and chemical potential energy, respectively.

### 3.2. Boundary Conditions

**Figure 3:**
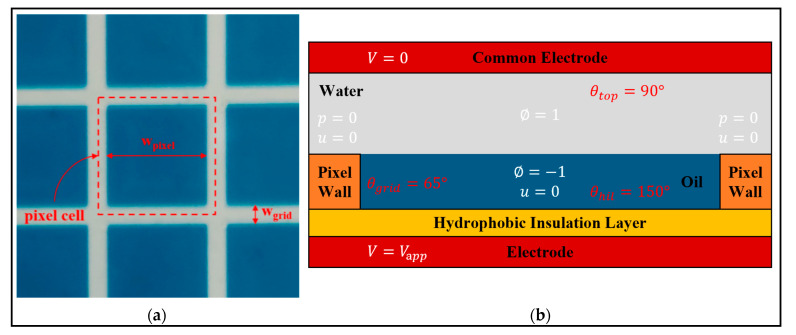
Pixel cell size and relevant boundary conditions: (**a**) The size of pixel cell. (**b**) The relevant boundary conditions.

The boundary conditions were the precondition that the governing equations had a definite solution on the boundary of the region [30]. The size of the pixel cell used in the calculation domain is shown in Figure 3a, and the relevant boundary conditions are shown in Figure 3b.

In the EWD structure model, zero-charge boundary conditions were set around the model, that is, n·D=0. This also meant that no displacement field could penetrate the boundary at the surrounding inner boundary, and the potential was discontinuous at the boundary. For electrostatic field boundary conditions, the potential Vapp and grounding boundary conditions should also be specified. And the wetted wall, initial interface, inlet, and outlet need to be set for the phase field boundary conditions. The wetted wall boundary conditions were determined by Equations (20) and (21).

(20) $$n\cdot \varepsilon^{2}\nabla \emptyset =\varepsilon^{2}\cos\theta_{w}\left|\nabla \emptyset \right|$$

(21) $$n\cdot \frac{\gamma \lambda }{\varepsilon^{2}}\nabla \psi =0$$

In the initial interface boundary condition, the two-phase flow contact interface was selected as the position of the initial interface, ∅=0. Both sides of the fluid of the EWDs were selected as the inlet and outlet boundary conditions, which were set as the outlet end in the actual simulation process, and the pressure constraint term p=0 was set at the end point. In addition, the initial value of volume force and velocity should be set, and the initial value of velocity was set to 0 (m/s). The detailed parameters of the proposed model were shown in Table 1.

## 4. Experimental Results and Discussion

The oil movement process in an EWD pixel cell was successfully simulated by the established simulation model. Therefore, based on this model, the influence of oil viscosity on the performance of EWDs can be investigated through experiments, including response time, maximum aperture ratio, and hysteresis effect. In experiments, the dynamic viscosity coefficient was used to characterize the oil viscosity, and the influence of oil viscosity on the hysteresis effect of the EWD was emphatically analyzed. All parameters were kept constant, and the temperature was kept at 25 °C. Segment function was used to describe the driving waveform, and the driving voltage ranged from 0 V to 30 V. The dynamic viscosity coefficient was dynamically changed, and the corresponding aperture ratio data were recorded.

According to Equation (1), the formula for calculating the aperture ratio in a two-dimensional model can be obtained. Assume that the pixel cell is square, and the side length is a. The contact surface between the oil and the hydrophobic insulating layer is round, with a diameter of d. Then, the calculation formula of the aperture ratio in the two-dimensional model can be obtained. The specific description is as follows, where $V_{R}$ is the rupture voltage:

(22) $$W_{A}\left(V\right)=\left\{\begin{array}{cc} 0, & V<V_{R}\\ \frac{4a^{2}-\pi d^{2}}{4a^{2}}\times 100\%, & \;\;V\geq V_{R} \end{array}\right.$$

### 4.1. Simulation of Oil Movement Process

**Figure 4:**
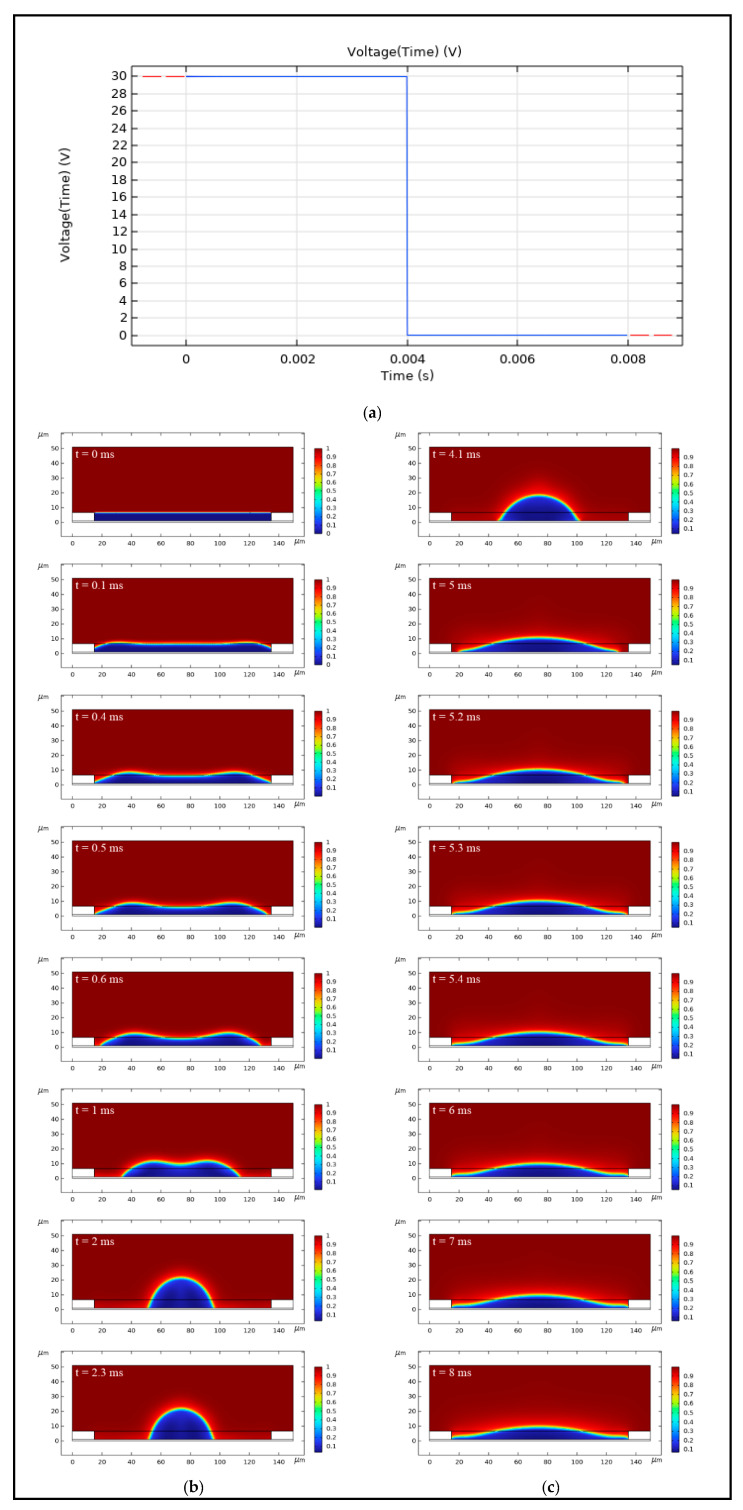
Simulation of oil movement process in an EWD pixel cell: (**a**) Driving waveform used in the simulation. The red line is the scale indicator line, and the blue line is the driving waveform line. (**b**) Pixel-on process. (**c**) Pixel-off process.

The oil movement process can be divided into an opening stage after applying the external voltage and a closing stage after removing the external voltage. A segment function was used to describe the traditional square wave, as shown in Figure 4a. Based on the established simulation model, the simulation of an entire oil movement process, including an opening stage and a closing stage, can be realized. As shown in Figure 4b,c, it was a simulation of the oil movement process in an EWD pixel cell. When t=0 ms, an external voltage of 30 V was applied, and the oil started to contract under the action of electric field force. At t=0.5 ms, the oil began to retract from the pixel wall, forming an exposed substrate region. At t=2.3 ms, the opening reached the maximum value. At this point, the oil opening process was completed. When t=4 ms, the applied external voltage was removed, and the oil began to spread under the effect of surface tension. When t=5.4 ms, the oil was closed and completely contacted the pixel wall. At this point, the oil closing process was completed.

### 4.2. Influence of Oil Viscosity on Response Time

**Figure 5:**
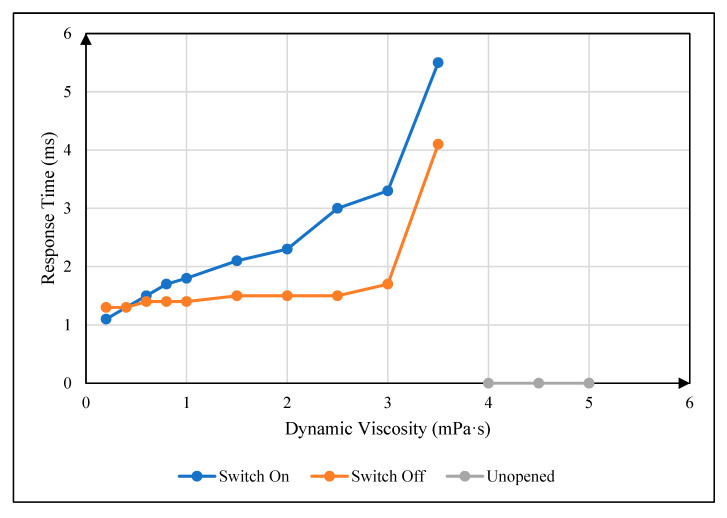
The relationship between oil dynamic viscosity coefficient and pixel response time. The blue curve is the pixel-on response time curve, the red curve is the pixel-off response time curve, and the green curve indicates that the pixel could not be opened within 16 ms.

In the experiment, a 30 V driving voltage was applied first and removed after the oil was completely opened. During this process, the pixel-on response time and the pixel-off response time were recorded when the oil was fully opened and fully closed under different oil dynamic viscosity coefficients. The relationship between the oil dynamic viscosity coefficient and pixel response time could be obtained, as shown in Figure 5. In the figure, the blue curve is the pixel-on response time curve, the red curve is the pixel-off response time curve, and the green curve indicates that the pixel could not be opened within 16 ms. It can be seen that the pixel-on response time and pixel-off response time increased with the increase in the oil dynamic viscosity coefficient. This was because the increase in oil dynamic viscosity would increase the viscosity resistance.

### 4.3. Influence of Oil Viscosity on Maximum Aperture Ratio

**Figure 6:**
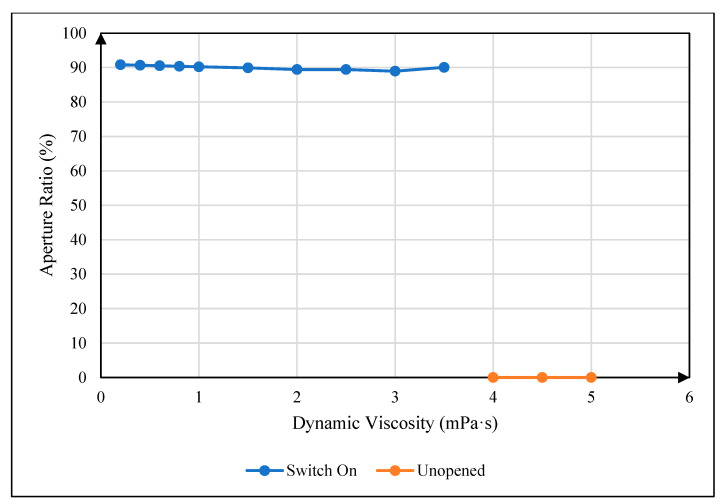
The relationship between oil dynamic viscosity coefficient and maximum aperture ratio. The blue curve is the maximum aperture ratio curve of a pixel, and the red curve indicates that the pixel could not be opened within 16 ms.

In the experiment, a 30 V driving voltage was applied. During this process, the maximum aperture ratio of a pixel cell under different oil dynamic viscosity coefficients was recorded when the oil was fully opened. The relationship between the oil dynamic viscosity coefficient and the maximum aperture ratio of a pixel cell could be obtained, as shown in Figure 6. The blue curve is the maximum aperture ratio curve of a pixel, and the red curve indicates that the pixel could not be opened within 16 ms. It can be seen that the maximum aperture ratio of a pixel cell was very close, indicating that the change in oil dynamic viscosity could not change the maximum aperture ratio of a pixel cell.

### 4.4. Influence of Oil Viscosity on Hysteresis Effect

**Figure 7:**
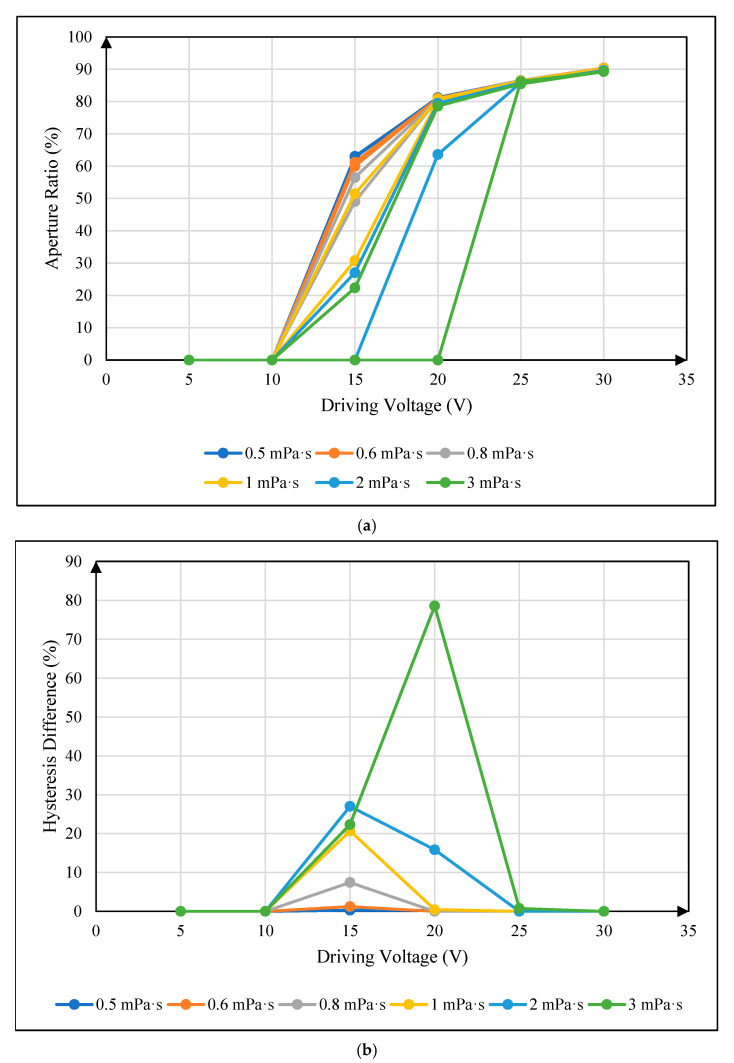
The relationship between oil dynamic viscosity coefficient and pixel hysteresis effect: (**a**) The relationship between oil dynamic viscosity coefficient and hysteresis curve. (**b**) The relationship between oil dynamic viscosity coefficient and hysteresis difference.

The dynamic viscosity coefficient of oil commonly used in the preparation of EWDs is 2 mPa∙s. In the experiment, the traditional square wave was used to drive a pixel cell, and the hysteresis curves under different dynamic viscosity systems were recorded. The relationship between the oil dynamic viscosity coefficient and the hysteresis curve could be obtained, as shown in Figure 7a. In order to more intuitively reflect the relationship between the oil dynamic viscosity coefficient and the hysteresis effect, the data of aperture ratio in the experimental results were further processed. The absolute value of the difference in the aperture ratio under the same voltage during the rising and falling process was defined as the hysteresis difference. The relationship between the oil dynamic viscosity coefficient and the hysteresis difference curve could be obtained, as shown in Figure 7b.

It can be seen from Figure 7 that during the movement of the oil in a pixel cell, the hysteresis difference first increases and then decreases with the increase in the driving voltage, forming a maximum hysteresis difference in this process, indicating the degree of hysteresis effect. The maximum hysteresis value increases with the increase in oil dynamic viscosity coefficient and decreases with the decrease in the oil dynamic viscosity coefficient, indicating that the oil dynamic viscosity coefficient is positively correlated with the hysteresis effect. This is because the increase in oil viscosity will cause an increase in shear stress during oil movement, that is, an increase in viscosity resistance. Greater viscous resistance will consume more electric field force to do work, resulting in a change in the effective work carried out by the same electric field force during oil movement.

To demonstrate the effectiveness of the proposed model, we compared and analyzed the experimental results with the conclusions of relevant research work, as shown in Table 2. This study primarily investigates the influence of oil viscosity trends on the performance of EWDs, laying a critical foundation for future experimental validation.

While this work focuses on first-principles modeling of viscosity-dependent hysteresis, emerging techniques in human–robot interaction (e.g., adaptive LLM control) and hybrid sensing systems (e.g., multimodal strain analysis) offer promising avenues for closed-loop optimization of EWDs in practical environments [32]. For example, combining simulations with real-time optical feedback to mitigate hysteresis.

## 5. Conclusions

In summary, based on the COMSOL Multiphysics simulation environment, the driving process of the hysteresis effect in an electrowetting display pixel cell was successfully simulated by coupling the laminar two-phase flow phase field and electrostatic field. Based on the proposed simulation model, the influence of oil viscosity on the hysteresis effect of electrowetting display was studied. Our simulations demonstrated that the maximum hysteresis difference in the hysteresis effect increased with the increase in oil viscosity and decreased with the decrease in oil viscosity. The oil viscosity had little effect on the maximum aperture ratio of EWD. The pixel-on response time and pixel-off response time increased with the increase in oil viscosity. Beyond electrowetting displays, the findings of this study can also be extended to other voltage-driven microfluidic systems. Although the simulation framework offers valuable insights, we acknowledge the following limitations: simplified boundary conditions, temperature dependence, and scalability. These extensions and limitations will be further explored in our future work.

## Figures and Tables

**Table 1:** Material, geometric, and interfacial parameters of the proposed model.

Parameters	Quantity	Symbol	Value	Unit
Material [31]	density of oil	$\rho_{oil}$	880	kg/m^3^
	density of water	$\rho_{water}$	998	kg/m^3^
	dynamic viscosity of oil	$\mu_{oil}$	0.002	Pa·s
	dynamic viscosity of water	$\mu_{water}$	0.001	Pa·s
	dielectric constant of oil	$\varepsilon_{oil}$	2.2	1
	dielectric constant of water	$\varepsilon_{water}$	80	1
	dielectric constant of hydrophobic insulating layer	$\varepsilon_{hil}$	1.95	1
	dielectric constant of pixel wall	$\varepsilon_{grid}$	3.28	1
Geometric [5]	width of pixel cell	$w_{pixel}$	150	μm
	height of pixel wall	$d_{grid}$	5.6	Μm
	width of pixel wall	$w_{grid}$	15	μm
	thickness of hydrophobic insulating layer	$d_{hil}$	1	μm
	thickness of oil	$d_{oil}$	5.6	μm
Interfacial [17]	surface tension of oil and water	$\gamma_{ow}$	0.015	N/m
	contact angle of pixel wall	$\theta_{grid}$	65	deg
	contact angle of hydrophobic insulating layer	$\theta_{hil}$	150	deg
	contact angle of top plate	$\theta_{top}$	90	deg

**Table 2:** Comparative analysis of experimental results on the influence of oil viscosity on the performance of EWDs.

Oil Movement Process	Response Time	Maximum Aperture Ratio	Hysteresis Effect
whole process	positive correlation	no correlation	positive correlation
whole process [31]	positive correlation [31]	-	positive correlation [14]

## Data Availability

All data are contained within the article.
